# Pelvic arteriovenous malformation causing per rectal haemorrhage – A case report

**DOI:** 10.1016/j.ijscr.2024.109291

**Published:** 2024-01-20

**Authors:** Yijun Gao, Suhrid P. Lodh, Nima Ahmadi

**Affiliations:** a St George Peritonectomy Unit, St George Public Hospital, Sydney, NSW 2217, Australia; b University of New South Wales, St George & Sutherland Clinical School, Sydney, NSW 2217, Australia

**Keywords:** Case report, Arteriovenous, Malformation, Gastrointestinal, Haemorrhage

## Abstract

**Introduction and importance:**

We present the case of a 17 years old girl with per rectal haemorrhage secondary to pelvic arteriovenous malformations (AVM) and potentially haemorrhoids. Pelvic AVMs are rare and extremely variable in their clinical presentation, size and location and pose a therapeutic challenge. Focus has turned towards interventional radiological procedures with angioembolisation as the main treatment form for pelvic AVMs.

**Case presentation:**

A 17 years old girl presented to a rural hospital with significant per rectal bleeding requiring transfer to a tertiary centre with interventional radiology capabilities. Diagnostic imaging determined the presence of a pelvic AVM as well as haemorrhoid. She had no prior history of haemorrhoids, per rectal bleeding or per vaginal bleeding. Further diagnostic imaging including a digital subtraction angiography and MRI pelvis was performed and her case was discussed at a multidisciplinary meeting where the decision was made for angioembolisation of a large right rectal AVM as well as precautionary banding of haemorrhoids that had developed secondary to outflow obstruction. A repeat CT mesenteric angiogram a month later demonstrated diminished appearances of the rectal AVM.

**Clinical discussion:**

Pelvic AVMs are a rare entity and are not a common cause for per rectal bleeding. There is currently no direct consensus on the optimum management of complex pelvic AVMs particularly those that present with a second pathology such as haemorrhoids. Surgical management often results in recurrence or rapid progression of the AVM lesion and recruitment of new blood supply further complicates the problem. Selective embolisation allows for control of haemorrhage and utilises chemical agents as well as detachable coils and balloons. However, postoperative pain and swelling can still be expected and multiple transcatheter embolisations may be required.

**Conclusion:**

The treatment of symptomatic pelvic AVMs is complex and requires a multidisciplinary approach with careful radiological planning prior to embolisation. Angioembolisation is becoming increasingly prevalent and multiple embolisation procedures may be required to reach the desired therapeutic effect.

## Highlights

• Arteriovenous malformations (AVMs) vary in location and size and present a difficult therapeutic challenge.
• Surgical management of pelvic AVMs is often inadequate and results in recurrence.
• Selective arterial embolisation utilises a variety of agents and is sufficient on its own to eliminate or improve symptoms.
• Multiple transcatheter embolisations may be required.
• The management of pelvic AVMs should be multidisciplinary involving surgeons as well as interventional radiologists.

## 1 Introduction

Congenital arteriovenous malformations (AVMs) result from one or more sites of abnormal direct communication between an artery and a vein without an intervening capillary bed. It is thought that they originate from abnormal endothelial cell proliferation and delayed vascular remodelling during foetal development [1]. These malformations can be extremely variable in their clinical presentation depending on their location and size and can present an extremely difficult therapeutic challenge. When symptoms develop, there may be pain, mass effect such as compression on adjacent organs, haemorrhage, or high output cardiac failure [2]. Pelvic AVMs typically involve the internal iliac artery, a branch from the common iliac artery, as the major feeding vessel [3]. Historically, both surgical and interventional radiological methods have been used to treat AVMs, with angioembolisation becoming more prominently utilised. The present case demonstrates the complexity of AVM management with the concurrent presence of haemorrhoids and the utility of selective angioembolisation as the main treatment for pelvic AVMs. This case report is in accordance with the SCARE checklist [4].

## 2 Case

A 17 years old female presented to a rural hospital with 2 days history of per rectal (PR) bleeding totalling 4 episodes. It was initially associated with defecation but then occurred with passage of flatus with an episode of 500 mL of estimated blood loss. On presentation she denied rectal pain and had some mild mid abdominal discomfort prior to the onset of PR bleeding. She had a history of constipation however denied any prior use of aperients. She had no history of previous PR bleeding or anaemia and denied previous per vaginal bleeding as she had an Implanon inserted a year prior. There was no relevant family history. On examination she was haemodynamically stable, her abdomen was soft with tenderness in the epigastrium and peri-umbilical region. A digital rectal examination was performed which revealed bright red blood on glove. Initial investigations demonstrated a haemoglobin of 13 g/dL, platelets of 247 and INR of 1.1.

**Fig. 1:**
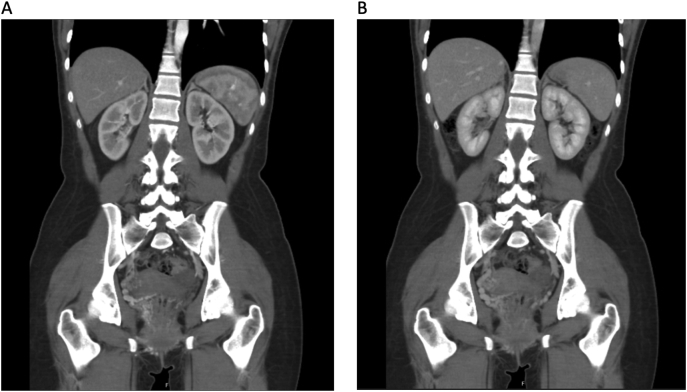
A: Coronal slice of the initial CTMA in the arterial phase demonstrating a right sided AVM. B: Coronal slice of initial CTMA in portal venous phase with no active gastrointestinal haemorrhage seen.

Following 3 further episodes of PR bleeding, she began to feel unwell and became tachycardic to 115 bpm with a blood pressure of 126/80 mmHg. Subsequent blood tests showed a drop of haemoglobin to 7 g/dL and she received transfusion with 2 units of packed red blood cells and a bolus infusion of 500 mL 0.9 % sodium chloride. A computed tomography mesenteric angiogram (CTMA) was performed which demonstrated prominent serpiginous vessels in the pelvis surrounding the vagina and involving the anterior aspect of the rectum with a right-sided predominance (Fig. 1). The findings in the arterial phase were highly suggestive of the presence of a pelvic AVM with no active gastrointestinal haemorrhage demonstrated.

**Fig. 2:**
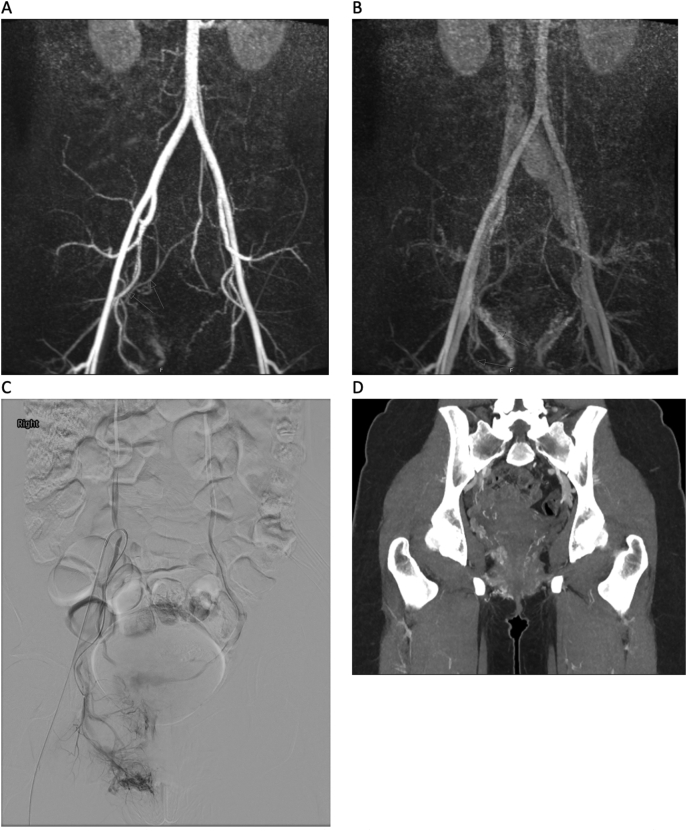
A: Coronal slice of her MRI pelvis demonstrating feeders from her right internal iliac artery as well as the inferior mesenteric artery and superior rectal artery. B: Draining veins were seen to predominantly course to the right internal iliac vein. C: DSA performed via right internal iliac artery with right sided haemorrhoids seen secondary to venous hypertension from outflow obstruction. D: Post embolisation CTMA demonstrating a diminished rectal component.

The decision was made for transfer to a tertiary level centre with interventional radiological capabilities. She remained tachycardic on arrival to the tertiary level centre with a blood pressure of 145/82 mmHg and a repeat haemoglobin of 10.6 g/dL. She underwent further investigation with magnetic resonance imaging of the pelvis which demonstrated a tangle of vessels in the right adnexal/pararectal region at approximately the level of the mid to lower rectum with feeding vessels identified from the right internal iliac artery. A few smaller feeding vessels were also identified from the inferior mesenteric and superior rectal arteries. The draining veins were predominantly seen coursing to the right internal iliac vein with a few traced into the left internal iliac vein. The vessels were seen to course the rectal wall on the right lateral aspect without definite luminal invagination. Prominent adnexal vessels were seen bilaterally attributing to background pelvic congestion. A digital subtraction angiography (DSA) was also performed and her case was discussed at the radiology multidisciplinary meeting. The right sided AVM appeared to be affecting the rectum (Fig. 2) and there appeared to be right sided haemorrhoids as a result of right venous hypertension secondary to outflow obstruction. The consensus recommendation for treatment was for angioembolisation as well as treatment of the haemorrhoid.

She underwent embolisation with access via the left common femoral artery. There was a small distal right superior rectal AVM with filling of a large right rectal haemorrhoid. This was microcanulated to within 1 cm of the fistula point and embolised with 0.1 mL of N-butyl cyanoacrylate (NBCA) lipiodol. A flexible sigmoidoscopy a few days later demonstrated no features of recent haemorrhage, and a proctoscopy visualised a moderate sized internal haemorrhoid at 7 o'clock which was banded without complications. She was discharged home the next day.

She had a repeat CTMA one month later which demonstrated prominent draining veins on the right side of the vagina extending superiorly to the level of the cervix and inferiorly to the level of the labia with presumably an underlying vascular malformation supplied by the internal iliac branches on the right. The rectal component had diminished compared to pre-embolisation (Fig. 2).

## 3 Discussion

Pelvic AVMs are rare and reported to affect less than 1 % of the general population with a female predominance [5]. They are a rare cause of PR bleeding compared to diverticular disease or haemorrhoids. Although we cannot comment with certainty the cause of this patient's PR bleeding, we suspect that her pelvic AVM was the main culprit. However, given the finding of a right sided haemorrhoid on imaging as well as on flexible sigmoidoscopy, the decision was made for rubber band ligation of the internal haemorrhoid as a prophylactic measure.

There is no direct consensus on the optimum management of complex pelvic AVMs. Proximal ligation of the feeding vessel is inadequate and will nearly always result in recurrence of the malformation which becomes more difficult to treat due to the recruitment of multiple new sources of blood supply to the lesion [3]. Incomplete surgical excision of the lesion or ligation of the inflow artery can lead to recurrence or rapid progression of the AVM lesion and make further endovascular treatment more difficult [6].

Selective arterial embolisation can be considered the first line of treatment for symptomatic AVMs and utilises agents such as N-Butylcyanoacrylate (NBCA) or ethylene vinyl alcohol polymer [7]. Detachable coils and balloons have a limited role as they provide proximal occlusion however, they are unable to reach the nidus [2]. Selective embolisation allows for control of haemorrhage and local or regional complications. However, the AVM nidus often remains and residual shunts are always present.

Embolisation or sclerotherapy has complication rates of nearly 10 % and can cause soft tissue and nerve injury through direct toxicity or by blockage of nearby microcirculation from the diffusion of the embolising agent [7]. Ethanol is an effective liquid embolic agent that directly damages the endothelium by acting as a protein denaturant. It is cost effective, but complications include significant oedema, skin necrosis, nerve damage, and dose-dependent pulmonary hypertension and cardiovascular collapse [8]. NCBA is a monomer acrylic glue able to form a cast of the vasculature that needs to be occluded and it polymerises on contact with blood to provide permanent occlusion and prevent replenishment through feeding branches [2]. It is preferred over ethanol in the context of AVMs with large draining veins or in the paediatric population where ethanol dosing needs to be limited [8]. Postoperative pain and swelling can still be expected and in this case, rectal ischaemia is a potential complication of the procedure.

A study by Jacobowitz et al. which is the largest series to date from a single institution on pelvic lesions suggested that transcatheter embolisation alone is sufficient to eliminate or improve symptoms in a high percentage of patients (29 of 35 patients, 83 %) [9]. Provided the high rate of recurrence, multiple transcatheter treatments may be required. Similarly, a recent retrospective study involving 93 patients reported 70–100 % effective devascularisation in 69 % of patients, which is in accordance with percentages presented in literature [10].

The pathogenesis of haemorrhoids remains multifactorial and includes factors such as hyperplasia of the arteriovenous network within the anorectal mucosa thereby resulting in increase in vascular pressure. Several studies have reported on the significance of the superior rectal artery as the main blood supply for the lower half of the rectum and the corpus cavernosus recti, the structural unit of the haemorrhoidal complex [11,12]. A case report by Komekami, Konishi [13] commented on an AVM in the rectum as the cause of haemorrhage from a patient's internal haemorrhoid. The presence of haemorrhoids is therefore not surprising in this case provided the presence of feeding vessels from the inferior mesenteric and right superior rectal artery as well as the venous hypertension from outflow obstruction. The decision was made for haemorrhoid banding during the flexible sigmoidoscopy as a prophylactic measure following her angioembolisation.

The management of pelvic AVMs should be multidisciplinary consisting of interventional radiology, vascular surgery and plastic surgery along with other surgical specialties depending on the location of the lesion. Patients should be monitored and followed up closely after treatment begins, with the potential for multiple embolisation procedures before significant clinical improvement is obtained or if further interventional treatment is precluded by anatomical or clinical considerations.

## 4 Conclusion

The treatment of pelvic AVMs remains a challenge and requires a multidisciplinary approach with careful radiological planning prior to intervention. Angioembolisation is becoming increasingly prevalent as the first and main approach to the treatment of pelvic AVMs and multiple procedures may be required prior to significant clinical improvement. Furthermore, a multi-modality approach may be required when more than one pathology is present.

## Consent

Informed consent for publication was provided by both the patient as well as her parent.

## Methods

The work has been reported in line with the SCARE criteria.

Sohrabi C, Mathew G, Maria N, Kerwan A, Franchi T, Agha RA; Collaborators. The SCARE 2023 guideline: updating consensus Surgical CAse REport (SCARE) guidelines. Int J Surg. 2023 May 1;109(5):1136–1140.

## Ethical approval

Ethics approval and consent were waived by the Human Research and Ethics Committee as the nature of the article did not appear to raise any ethical risks.

## Funding

This study did not receive any specific grants from public, commercial or not-for-profit funding agencies.

## Guarantor

Yijun Gao.

## CRediT authorship contribution statement

Yijun Gao – data collection and analysis, writing the paper.
Nima Ahmadi – contributor and editor.
Suridh Lodh – contributor and editor.

## Declaration of competing interest

The authors state no conflict of interest.
